# Is it valid to assess an individual’s performance in team training simulation when the supporting team are confederates? A controlled and randomized clinical trial

**DOI:** 10.1186/s12909-022-03747-3

**Published:** 2022-09-19

**Authors:** Jérémie Traoré, Frédéric Balen, Thomas Geeraerts, Sandrine Charpentier, Xavier Dubucs, Charles-Henri Houzé-Cerfon

**Affiliations:** 1 Department of Emergency Medicine, Toulouse University Hospital, Toulouse, France; 2 Department of Anesthesiology and Intensive Care Medicine, Toulouse University Hospital, Toulouse, France; 3 Toulouse Institute of Simulation Healthcare (Institut Toulousain de Simulation en Santé, ItSimS), University Hospital Toulouse, Toulouse, France; 4 University Toulouse III Paul Sabatier, Toulouse, France; 5 UMR EFTS (Education, Formation, University of Toulouse 2 Jean Jaurès, Savoirs), Toulouse, Travail France

**Keywords:** Health education, Simulation training, Interprofessional education, Crisis resource management, Emergency medicine

## Abstract

**Background:**

During simulation training, the confederate is a member of the pedagogical team. Its role is to facilitate the interaction between participants and the environment, and is thought to increase realism and immersion. Its influence on participants' performance in full-scale simulation remains however unknown. The purpose of this study was to observe the effect of the presence of confederates on the participants’ performance during full-scale simulation of crisis medical situations.

**Methods:**

This was a prospective, randomized study comparing 2 parallel groups. Participants were emergency medicine residents engaging in a simulation session, with or without confederates. Participants were then evaluated on their Crisis Resource Management performance (CRM). The overall performance score on the Ottawa Global Rating Scale was assessed as primary outcome and the 5 non-technical CRM skills as secondary outcomes.

**Results:**

A total of 63 simulation sessions, including 63 residents, were included for statistical analysis (*n* = 32 for Control group and 31 for Confederate group). The mean Overall Performance score was 3.9 ± 0.8 in the Control group and 4.0 ± 1.1 in the Confederate group, 95% confidence interval of the difference [-0.6; 0.4], *p* = 0.60. No significant differences between the two groups were observed on each CRM items (leadership, situational awareness, communication, problem solving, resource utilization)

**Conclusion:**

In this randomized and controlled study, the presence of confederates during full-scale simulated practice of crisis medical situations does not seem to influence the CRM skills performance of Emergency medicine residents.

**Trial registration:**

This study does not need to be registered on Clintrial as it does not report a health care intervention on human participants.

**Supplementary Information:**

The online version contains supplementary material available at 10.1186/s12909-022-03747-3.

## Background

A fundamental assumption of simulation is that learning is enhanced when the environment seems realistic. Full-scale simulation attempts to recreate every elements of a real-life situation in order to persuade the participants to accept the “fiction contract” and to become fully engaged in the simulation experience [1–3]. Perceived realism is linked to the interactions between the participant and the environment. The use of a confederate in a full-scale simulation is of common practice to facilitate these interactions and enhance the immersion of the participant in the environment. The confederate is not a learner, but a member of the pedagogical team with a scripted role during the simulated practice phase [4]. He aims to provide realism, challenges or information when they cannot be transcribed by the simulator [5, 6]. The confederate is used to improve the realism by limiting the biases induced by the simulated environment.

Experts suggest using full-scale simulation to enhance interprofessional teamwork and to deepen Crisis Resource Management (CRM) skills, as leadership, communication, problem solving, resources management and situational awareness [7]. The lack of CRM skills in the management of critical situations has been proven to be associated with adverse events and malpractice cases [8, 9]. Learning CRM skills leads to better safety in patient outcomes, including a decrease in mortality [10].

The main limitation to the implementation of full-scale simulation in medical education programs is human resources [11]. Appropriate and justified supervision is therefore essential [12–14]. The influence and role of each member of the pedagogical team should be argued, considering the performance of the participants as the main outcome. To our knowledge, the effects of confederate presence on the performance of participants in a full-scale simulation has not been addressed in proper quality studies.

The purpose of this study was to observe the effect of the presence of confederates on the participants’ performance during full-scale simulation of crisis medical situations.

## Methods

### Study design

This randomized controlled study took place in a French university medical simulation center from December 2018 to February 2020. According to the French ethic and regulatory law, article R1121-1 of the public health code, ethical approval for the study was waived by the national ethical committee (CPP, Comité de Protection des Personnes). It was registered at the register of epidemiologic studies of Toulouse University Hospital (RnIPH 2019–53) and has also been declared to National Commission of Informatics and Liberty (CNIL number: 2206723 v0). The University Hospital signed a commitment of compliance to the reference methodology MR-004. All participants provided written informed consent to participate.

### Participants

Participants were residents within the 2^nd^ year, 3^rd^ year or 4^th^ year post-graduate of Emergency Medicine at Toulouse University. In the curriculum of a resident in Emergency Medicine in Toulouse University, a 2^nd^ year participates to 2 simulation of crisis medical situation within the year, 4 sessions for a 3^rd^ year and a 4^th^ year. The criterion for non-inclusion was the resident's refusal to participate in the study. Each resident participated in a single simulation session as part of the study.

### Study protocol

The planning of the simulation sessions was fully integrated into the curriculum of the Emergency Medicine residents according to a simulation training program [15]. Each session was developed in accordance with the French National Health Authority (Haute Autorité de Santé).

The session was led by one simulation instructor and one medical expert. The instructors belong to a group of 9 instructors (7 from a University Hospital and 2 from a General Hospital, including 2 nurses and 7 physicians) with a university degree in simulation training. The medical experts are emergency physicians (*n* = 15) with at least 5-year experience and specific knowledge in pediatrics, advanced life support, obstetrics, resuscitation, and airway management. The overall purpose of these sessions was to learn communication skills, knowledge of respective roles (leadership/fellowship), involvement in shared decision-making and team coordination.

A simulation session was intended for a complete medical team composed of an emergency physician (resident), a nurse and a medical support worker.

Each simulation session lasted 1 h and was divided into 3 parts: briefing (15 min), simulated practice (15 min) and debriefing (30 min). The briefing prepared the team for the simulated practice, created a positive learning environment, encouraged the emotional security and introduced the clinical situation. Debriefing was conducted by the assessors according to the RUST model and the good-judgement practice [16].

In the simulation, participants' performance was based on the assessment of CRM skills, a set of non-technical skills required to manage medical emergencies [17].

This assessment was carried out immediately after the debriefing by the simulation instructor and the expert physician who conducted the debriefing and were trained in the use of this assessment grid. The assessors (medical expert and instructor) were randomly assigned to each simulation session considering their availability. The participants were assessed individually by the assessors. Then results were shared among assessors in order to find a consensus on the final scoring.

Participant demographics were collected using a paper questionnaire to be completed before the simulation session. This data was then transferred to an anonymized Excel file.

In the Confederate group, two confederates playing the role of a nurse and a medical support worker were part of the session. The confederates are familiar with the simulated environment. They were asked to take no initiatives and to wait for the participant instructions without guidance. The role assigned to the confederates was to play their professional role (nurse emergency or medical support worker) and to help learners work in an unfamiliar simulation environment, guide learners to meeting learning objectives (for example, if the learner did not realize the patient was having a severe blood loss, the confederate could ask: “do you want me to do a hemoglobin test?”), protect simulators from damage, increase learners’ engagement in the scenario by selectively increasing participants’ cognitive load (by taking no initiative without a formal demand from the leaner (i.e. the physician) enrolled in the simulation), communicate with ‘‘control room’’ during scenarios (through an earpiece), offer insider experience during debriefing and/or evaluation, standardize manner in which information is conveyed to study participants (e.g., laboratory data, physical signs and symptoms, whether the patient has a known allergy, and so forth) to limit variability, minimizing risk for bias [5].

In the *Control group*, there was no confederate. An interprofessional group composed of a nurse and a medical support worker from the Emergency Department which were operating during the simulated practice but unfamiliar with the scenarios beforehand. They were asked to act as health professionals just as they would in a real-life situation. They could take initiatives and suggest therapeutics. The “real” team member wore earpiece, was briefed prior to scenario they will be asked to convey clues and then was clued by instructors at appropriate times/instructors phone into room with “results” at specified points.

The professionals of the *Confederate group* and the *Control group* both work at the pre-hospital medical service (SAMU) of the CHU of Toulouse. They have the same pre-requirement and clinical experience of at least 5 years of practice. Participants did not know the professionals in advance and could not identify whether they were confederates or not.

### Scenarios used in the study

The scenarios were created by the different pedagogical managers of the simulation centers, in collaboration with the medical and paramedical trainers of each discipline. Each scenario was validated according to a standardized grid by the research team, composed of the university program director and simulation teachers (Additional file 1: Appendix A). They all have academic qualification in medical education.

It made it possible to evaluate the authenticity through the characteristics of a "complex problem" and the relevance of the professional situation described to promote learning to manage a critical situation in an interprofessional team.

Their design took into account the possibility of adapting the scenario on certain aspects related to environmental specificities linked to the learners' working conditions (emergency room, pre-hospital environment, ward…). Several professional situations were modelled in order to expose teams to various crisis situations such as cardiac arrest, difficult airway management, traumatic shock (Additional file 2: Appendix B).

The scenarios were performed in the emergency room or pre-hospital training room simulated of Toulouse Institute of Health Simulation center. The simulation laboratory was configured as an emergency room or prehospital setting (living room or bedroom) with a full-body manikin patient simulator (SimMan 3G; Laerdal Medical, Stavanger, Norway). The essential equipment were provided exactly as in real life, with a patient monitoring, a defibrillator, a resuscitation trolley or bag. Participants were briefed about the equipment before the beginning of the simulation session.

The choice of scenario was made randomly by the assessors at the beginning of the session according to their year of residency of each student.

### Confederates

Confederates were health professionals of the Emergency Department of the University Hospital. They have all benefited from a training-course in medical education. This is a five-day training course in order to master active teaching techniques, to integrate the specificities of the professional context into the practice of emergency care and to harmonize evaluation techniques. Within this framework, they were also trained in the use of full-scale simulation and specifically in the role of confederate during a three-day training course. They participated to debriefing as their own professional role. They did not take part in the assessment of the participants. Thirteen confederates participated in the study out of 24 trainers available at the Emergency department (Table 1). They were selected on a voluntary basis and according to their availability.

**Table 1:** Confederates’ demographic characteristics (Confederate group)

Demographic Characteristics	Confederates (*n* = 13)
Mean age ± SD	44.7 ± 4.2
Gender, n (%)	
Female	7 (54)
Professional Category, n (%)	
Medical support worker	6 (46)
Nurse	7 (54)
Years of work experience, mean ± SD	18.8 ± 4.3
Years of experience as a trainer, mean ± SD	6.5 ± 3.7
Number of simulation sessions/year/trainer, mean ± SD	27.7 ± 24.2
Number with more than 25 simulation sessions/year, n (%)	5 (38)

### Assessors

The assessors (medical expert and instructor) had at least 3 years' experience in CRM course. They had undergone training to use Ottawa GRS and use it frequently. The participants were assessed individually by each assessor. Then results were shared among assessors in order to find a consensus on the final scoring. Instructor and medical expert were not aware of the research hypothesis and outcomes of the study.

### Rating scale

At the end of the simulation session, the resident was assessed on CRM performance according to the Ottawa Global Rating Scale (OGRS), composed of five CRM-related domains (leadership, communication, problem solving, resource utilization, situational awareness) and an overall performance rating. Each domain was scored on a seven-point Likert-like response format (7 being the highest), with descriptive anchors to aid to scoring (Additional file 3: Appendix C). The overall performance rating is an independent criteria. The inter-rater measurement difference in this evaluation grid is very small (Intraclass Correlation Coefficient > 0.6 interpreted as good according to Cicchetti) [18]. This scale is a good instrument in simulated crisis situation assessment with a good Inter CC score (α = 0.96), and a good user-friendly, which is important in a complex assessment [19]. It has shown strong interrater reliability and discriminative ability among postgraduate physician trainees [20].

### Outcomes

The primary outcome for assessing the performance of the participants was the average scores obtained by the residents in each group (Control and Confederate) on the OGRS Overall Performance during the simulated practice.

The secondary outcomes for assessing each CRM performance of the residents were the average scores obtained by participants in each group (Control and Confederate) on 5 skills: leadership, communication, problem solving, resource utilization and situational awareness.

### Randomization

**Fig. 1:**
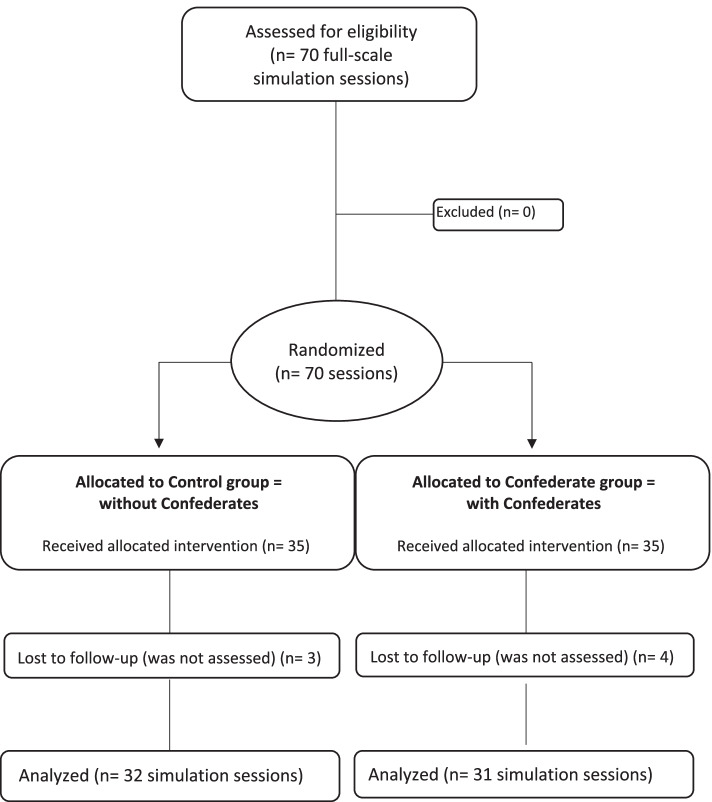
Study flow

A planning of the simulation sessions on the study inclusion period had been prepared. The randomization of the sessions was realized by the Clinical Research Unit at Toulouse University Hospital in a 1:1 ratio, into 2 groups: Control group and Confederate group. The confederates were attributed to each session according to the randomization. No sessions were excluded prior to randomization (Fig. 1).

### Statistical analysis

The number of simulation sessions was determined on the primary assumption that participants in the Confederate group would have a 0.4 point variability on the OGRS Overall Performance, compared to participants in the Control group [21]. Based on Cohen's definition of statistical analyses in behavior science, a standard deviation of 0.5 was used [22]. Given the initial hypothesis, a two-tailed test with a power of 80% was used. With these parameters, the number of simulation sessions required is a minimum of 50 sessions, i.e. two groups of 25 sessions.

Statistical analysis of the anonymized data was performed using BiostaTGV® software. All study variables were analyzed individually, checking for missing data and outliers. No imputation method was used for missing values.

The distribution of the quantitative variables was represented by the mean followed by the standard deviation after checking for normality. The comparative analyses were performed using the Student's test after checking for application criteria (distribution of values according to a Gaussian Normal Law). The threshold of statistical significance allowing to reject the H0 hypothesis, according to which the means are equal in the two groups, was considered reached when the risk of error was less than 5% (*p*-value < 0.05).

## Results

### General characteristics of the participants

Sixty-three simulation sessions, including 63 residents, were included for analysis: 32 for Control group and 31 for Confederate group (Table 2). No student refused to participate to the study. The residents’ demographic characteristics were homogeneous between the two groups.

**Table 2:** Characteristics of the participating residents

	**Control group** ***N* = 32**	**Confederate group** ***N* = 31**
**Age (median ± SD)**	26.5 ± 3.25	26.9 ± 2.83
**No. Women (%)**	19 (59)	19 (61)
**Semester of Residency (%)**		
No. 3^rd^ semester	15 (48)	20 (64)
No. 5^th^ semester	5 (15)	5 (16)
No. 7^th^ semester	12 (37)	6 (20)
**Simulation Theme (%)**		
Pediatrics	14 (44)	10 (33)
Cardiac Arrest	7 (22)	12 (38)
Cardiology	6 (19)	4 (13)
Vital Medical Emergencies	5 (15)	5 (16)
**No. of Previous Full-Scale Simulation Sessions (%)**		
0	17 (53)	17 (55)
1	10 (31)	9 (29)
2	5 (16)	5 (16)

Primary outcome (Table 3).

**Table 3:** The CRM performance scores of the participants in the Control and Confederate group

	**Control group**	**Confederate group**	**Difference between groups (IC95%)**	p-value*
Overall Performance	3.9 ± 0.8	4.0 ± 1.1	-0.12 [-0.61; 0.35]	*0.60*
Leadership	4.7 ± 0.9	4.2 ± 1.1	0.1 [-0.43; 0.63]	*0.71*
Situational Awareness	4.5 ± 1.0	3.9 ± 1.2	0.54 [-0.02; 1.09]	*0.059*
Communication	4.6 ± 0.7	4.5 ± 1.1	0.08 [-0.38; 0.54]	*0.73*
Problem Solving	4.1 ± 1.1	4.4 ± 1.1	-0.25 [-0.82; 0.34]	*0.40*
Resource Utilization	4.4 ± 0.7	4.3 ± 1.2	0.12 [-0.38; 0.61]	*0.64*

*Results are expressed as mean* ± *SD*

^*^Results were considered significant if p-value < *0.05 and Confidence Interval excluded 0*

The mean Overall Performance score was 3.9 in the Control group and 4.0 in the Confederate group, 95% confidence interval of the difference [-0.6; 0.4], *p* = 0.60.

Secondary outcomes (Table 3).

No significant differences between the two groups were observed on each CRM items (leadership, situational awareness, communication, problem solving, resource utilization).

## Discussion

In this randomized and controlled study, the presence of confederates during full-scale simulated practice of crisis medical situations does not seem to improve the CRM skills performance of Emergency medicine residents.

In the literature, it has been shown that performance and learning depend on the immersion quality into the simulated environment [23]. To ensure participant’s immersion, standards of best practice for simulation design expressly require to use confederates who have formal training in simulation-based education. The facilitative approach must also be predetermined during the design phase of the scenario and the level of confederates’ involvement must be inversely proportional to the participant’s knowledge and experience [24]. Our randomized controlled study suggests that using confederates with formal training has no added value on the participant’s performance. Indeed, situational awareness, problem solving and resource utilization scores showed no significant difference between the two groups. The confederates do not seem to have more effect on residents’ CRM performance than nurse and/or medical support worker without formal training in simulation-based education or predetermined facilitative approach. Our results do not show that the confederates improve resident’s immersion and interaction with the simulated environment enough to influence their performance.

In this study, within the Control group, nurses and medical support workers were not informed beforehand of the outcome of the scenario enabling a spontaneous attitude as a real life. The learning objective were team management in crisis situations. We could think that the interactions between team members is essential for the realism and impact the CRM performance. The nurses and medical support workers have been probably more focus on their role as healthcare professionals and allowed more realistic interactions with the resident.

Research suggests that in a full-scale simulation, participant’s behavior is closely linked to his confederate’s one so that he comes to imitate their actions [25]. In the case of confederates with very precise scripts often far from reality, deliberately less "helpful" to increase the difficulty of a simulation session, it can be assumed that the simulation session would be locked into a sterile situation without participation or mutual support [26]. In our study, in the Confederate group, confederates were given instructions to act less helpful by taking no initiatives even though they probably would in a real-life situation. By being part of the pedagogical team and knowing the scenarios outcomes beforehand, it could be hypothesized that their involvement within the simulation was less intense especially with less emotional participation such as doubt or stress and decreased the interaction between the participants and the confederates. However, the communication skill scores showed no significant differences between the two groups. Suggesting that the presence of trained confederates has no negative impact on participants’ CRM performance. Nevertheless, the standardization of confederates’ behavior in full-scale simulation could prevent some errors of the team during the trained session and prevent them from being addressed during the debriefing. According to our results, confederates do not seem to limit the biases related to the simulated environment that could influence the performance of the participants [24]. The development of training team-based simulation is challenged by the availability of pedagogical staff and by the limited number of learners in each session [27].

As the two groups were only compared on their CRM performance in a simulated scenario which has poor significance in a real life setting, it would be interesting to assess for a further work the effects if any, of confederate/non-confederate on learning and subsequent transfer to the clinical environment.

### Limitations

One of the main limitations of this study is the lack of blinded and compared assessment. Assessment was performed by a group of instructors who undergone training to use the OGRS and they use it frequently. The use of the OGRS has been shown to have a good reproducibility between observers but the study design did not allow to have a blinded nor a compared assessment [19]. Furthermore, assessor distribution was not random but according to their availability. It would have strengthen the design if assessors assessed similar numbers of scenarios across similar year groups of participants.

Another limitation of the study concerns the differences in the participants’ semester of residency between the two groups. Despite randomization, more 7^th^ semester residents were present in the Control group and more 3^rd^ semester residents in Confederate group. This could lead to a difference in performance in favor of control group. Nevertheless, this difference is relatively modest and probably insufficient to impact the CRM performance of either group. Moreover, the experience for simulation tool use was similar between groups. This have been shown to be an import impact on performance [28].

## Conclusion

The presence of confederates during the simulated practice of a full-scale simulation seems to have no significant impact on the participants’ CRM performance.

This study questions the interest of confederates in simulation-based education. Not using confederates could reduce the pedagogical staff mobilized in full-scale simulation and improve the implementation of interprofessional simulation but still preserve participants’ performances. The impact of confederates’ presence during the debriefing phase has not been studied by the present study and should eventually be explored in further studies.

## Supplementary Information

**Additional file 1: APPENDIX A.** SCENARIO ASSESSMENT GRID.

**Additional file 2: APPENDIX B.** SCENARIOS.

**Additional file 3: APPENDIX C.** OTTAWA GLOBAL RATING SCALE.

## Data Availability

The datasets used and/or analyzed during the current study are available from the corresponding author on reasonable request. All authors confirm that all methods were carried out in accordance with relevant guidelines and regulations.

## Abbreviations

CRM: Crisis Resource Management
OGRS: Ottawa Global Rating Scale
