# Supplementary material for: Is it valid to assess an individual’s performance in team training simulation when the supporting team are confederates? A controlled and randomized clinical trial
Source: BMC Med Educ. 2022 Sep 19;22:685. doi: 10.1186/s12909-022-03747-3 (PMC9487079; doi:10.1186/s12909-022-03747-3)
Supplement: Supplementary file 1 — Additional file 1: APPENDIX A. SCENARIO ASSESSMENT GRID. [file 12909_2022_3747_MOESM1_ESM.docx]

APPENDIX A – SCENARIO ASSESSMENT GRID

Simulation center name:

Scenario number:

Field of care (emergency, intensive care, pediatrics, obstetrics…):

Care environment (home, operating room, birth room):

The scenario precisely described:

|  | YES /NO | If "no", comments |
| --- | --- | --- |
| The care environment:  vital emergency room, treatment room, operating room, pre-hospital environment (public road, car...), birth room, delivery room, ... |  |  |
| Equipment and external resources available (drugs, respirator, medical equipment, imaging results, biological results, ...) |  |  |
| The various stakeholders (nurse, care assistant, physicians, technicians, operating room nurse, nurse anesthetist, ...). |  |  |
| The clinical situation corresponds to a so-called "critical situation". |  |  |
| The situation described requires the involvement of all members of an interprofessional team. |  |  |
| Not all the clues needed to solve the problem are immediately available. |  |  |
| The problem evolves in the course of its investigation |  |  |
| All timeframes are described and are compatible with real-life timeframes (examination requests, biological results, completion of tasks, observation of the effect of a therapy). |  |  |
