# Supplementary material for: Is it valid to assess an individual’s performance in team training simulation when the supporting team are confederates? A controlled and randomized clinical trial
Source: BMC Med Educ. 2022 Sep 19;22:685. doi: 10.1186/s12909-022-03747-3 (PMC9487079; doi:10.1186/s12909-022-03747-3)
Supplement: Supplementary file 2 — Additional file 2: APPENDIX B. SCENARIOS. [file 12909_2022_3747_MOESM2_ESM.docx]

APPENDIX B – SCENARIOS

| Themes | Scenario titles |
| --- | --- |
| Cardio Respiratory Arrest | - Adult patient presenting cardiac arrest due to acute coronary artery disease in the Emergency Department. - Adult patient presenting cardiac arrest due to thromboembolic disease in the Emergency Department. - Adult patient presenting cardiac arrest at home due to coronary artery disease with refractory rhythm disorder. - Adult patient presenting cardiac arrest at home due to drug intoxication. |
| Pediatrics | - Child presenting cardiac arrest in a pre-hospital drowning setting. - Child presenting obstructive respiratory distress at home. - Child presenting respiratory distress of laryngeal origin at home. - Child presenting severe head injury at home. - Child presenting epileptic seizure disorder in a hospital setting. - Child presenting unexpected infant death at home. |
| Vital Medical Emergencies | - Adult patient presenting severe rhythm-related discomfort in pre-hospital care. - Adult patient presenting acute heart failure in the Emergency Department. - Adult patient presenting hemorrhagic shock in the Emergency Department. - Adult patient presenting septic shock in the Emergency Department. |
| Cardiology | - Adult patient presenting ST+ coronary syndrome in the Emergency Department. - Adult patient presenting non-ST coronary syndrome in the Emergency Department. - Adult patient presenting non-tolerated pre-hospital ventricular tachycardia. - Adult patient presenting a Bouveret's disease crisis in pre-hospital. - Adult patient presenting hypertensive acute pulmonary edema in pre-hospital. - Patient with acute pulmonary edema with cardiogenic shock in the Emergency Department. |
