# Supplementary figures and images for: Is it valid to assess an individual’s performance in team training simulation when the supporting team are confederates? A controlled and randomized clinical trial
Source: BMC Med Educ. 2022 Sep 19;22:685. doi: 10.1186/s12909-022-03747-3 (PMC9487079; doi:10.1186/s12909-022-03747-3)

APPENDIX C - OTTAWA GLOBAL RATING SCALE

**
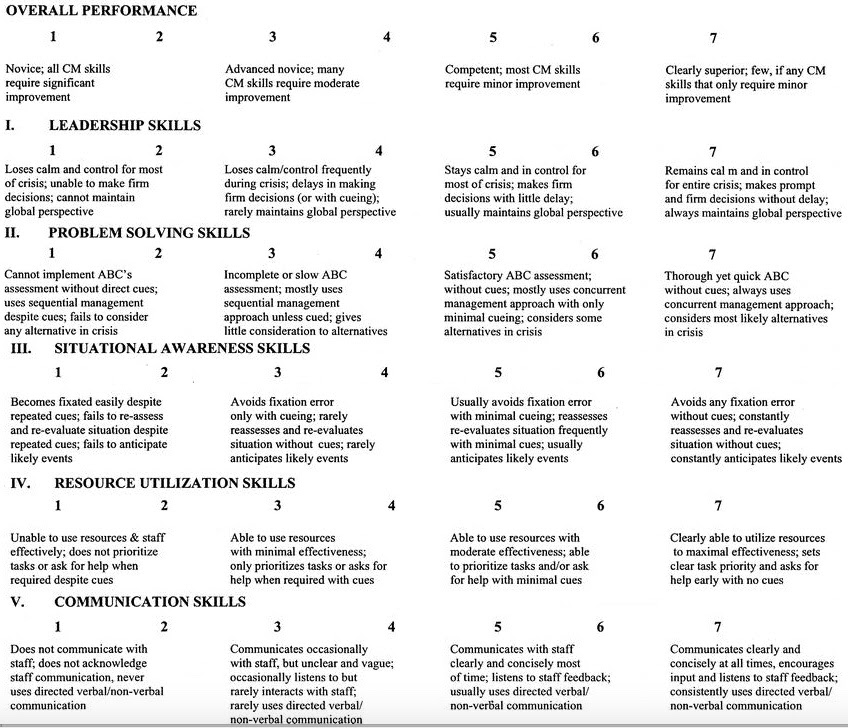
**

Supplement: Supplementary file 3 — Additional file 3: APPENDIX C. OTTAWA GLOBAL RATING SCALE. [file 12909_2022_3747_MOESM3_ESM.docx]
